# GENCODE: producing a reference annotation for ENCODE

**DOI:** 10.1186/gb-2006-7-s1-s4

**Published:** 2006-08-07

**Authors:** Jennifer Harrow, France Denoeud, Adam Frankish, Alexandre Reymond, Chao-Kung Chen, Jacqueline Chrast, Julien Lagarde, James GR Gilbert, Roy Storey, David Swarbreck, Colette Rossier, Catherine Ucla, Tim Hubbard, Stylianos E Antonarakis, Roderic Guigo

**Affiliations:** 1 Wellcome Trust Sanger Institute, Wellcome Trust Campus, Hinxton, Cambridge CB10 1SA, UK; 2 Grup de Recerca en Informatica Biomedica, Institut Municipal d'Informatica Medica-Universitat Pompeu Fabra, Pg. Maritim de la Barceloneta, 08003 Barcelona, Catalonia, Spain; 3 Department of Genetic Medicine and Development, University of Geneva Medical School and University Hospitals of Geneva, Geneva, Switzerland; 4 Center for Integrative Genomics, University of Lausanne, Lausanne, Switzerland; 5 Centre de Regulacio Genomica, Pg. Maritim de la Barceloneta, 08003 Barcelona, Catalonia, Spain

## Abstract

**Background:**

The GENCODE consortium was formed to identify and map all protein-coding genes within the ENCODE regions. This was achieved by a combination of initial manual annotation by the HAVANA team, experimental validation by the GENCODE consortium and a refinement of the annotation based on these experimental results.

**Results:**

The GENCODE gene features are divided into eight different categories of which only the first two (known and novel coding sequence) are confidently predicted to be protein-coding genes. 5' rapid amplification of cDNA ends (RACE) and RT-PCR were used to experimentally verify the initial annotation. Of the 420 coding loci tested, 229 RACE products have been sequenced. They supported 5' extensions of 30 loci and new splice variants in 50 loci. In addition, 46 loci without evidence for a coding sequence were validated, consisting of 31 novel and 15 putative transcripts. We assessed the comprehensiveness of the GENCODE annotation by attempting to validate all the predicted exon boundaries outside the GENCODE annotation. Out of 1,215 tested in a subset of the ENCODE regions, 14 novel exon pairs were validated, only two of them in intergenic regions.

**Conclusion:**

In total, 487 loci, of which 434 are coding, have been annotated as part of the GENCODE reference set available from the UCSC browser. Comparison of GENCODE annotation with RefSeq and ENSEMBL show only 40% of GENCODE exons are contained within the two sets, which is a reflection of the high number of alternative splice forms with unique exons annotated. Over 50% of coding loci have been experimentally verified by 5' RACE for EGASP and the GENCODE collaboration is continuing to refine its annotation of 1% human genome with the aid of experimental validation.

## Background

The complete sequence of the euchromatic region of the human genome provides a new opportunity to establish the complete catalogue of the human genes. Although automated gene prediction has improved greatly over the years and the human gene count is thought to be between 20,000 and 25,000 protein-coding genes [1], defining a gene is not a trivial issue. According to classic genetics, genes are inheritable units responsible for an associated phenotype. Although in some cases this relationship derives from mutation of non-coding DNA or regulatory elements, in most cases it is synonymous with protein-coding genes. However, in the past four years there has been an explosion in the discovery of transcripts with no apparent coding potential (termed non-coding RNAs) and there are indications these could play as important a role in cellular function as proteins [2,3].

**Figure 1:**
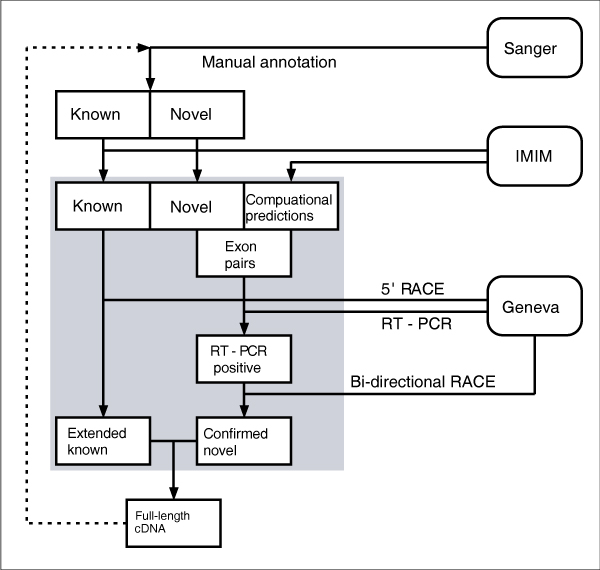
The GENCODE pipeline. This schematic diagram shows the flow of data between the three groups involved in the GENCODE consortium (HAVANA, IMIM and Geneva) to produce an experimentally verified annotation of the ENCODE region.

In an effort to investigate and understand all the functional elements in the human genome, the ENCODE project (Encyclopedia of DNA Elements) [4] was established. In this pilot stage, the aim of the ENCODE project is to investigate in great depth, computationally and experimentally, 44 regions totaling 30 Mb of sequence representing approximately 1% of the human genome. As part of this project, the GENCODE consortium [5] was formed to identify and map all protein-coding genes within the ENCODE regions. This is achieved by a combination of initial manual annotation by the HAVANA team [6], experimental validation by the GENCODE consortium, and a refinement of the annotation based on these experimental results (Figure 1).

This annotation is used as a reference set by all the ENCODE consortium members. It also represents the standard to which the automated prediction programs were assessed during the ENCODE Genome Annotation Assessment Project (E-GASP) 05 workshop (see [7] in this issue). This report describes how the manual annotation and experimental verification were performed. It also highlights some interesting features in the GENCODE annotation and indicates the weaknesses of the automated predictions compared to the manual annotation.

## Results and discussion

### Initial classification of loci

The HAVANA group divides gene features into different categories of which only the first two (known and novel coding sequence (CDS)) are confidently predicted to be protein-coding genes. The common factor between all annotated gene structures is that they must be supported by transcriptional evidence, through homology to cDNA, expressed sequence tags (ESTs) and/or protein sequences. The following are the gene types first applied to the human chromosome 20 annotation [8] and later expanded to fully classify the annotation produced for the ENCODE project.

#### Known genes

Known genes are identical to human cDNA or protein sequences and identified by a GeneID in Entrez Gene [9].

#### Novel coding sequence

Novel coding sequences have an open reading frame (ORF) and are identical, or have homology, to cDNAs or proteins but do not fall into the above category; these mRNA sequences are submitted to public databases, but they are not yet represented in Entrez Gene or have not yet received an official gene name from the nomenclature committee [10]. They can also be novel in the sense that they are not yet represented by an mRNA sequence in the species concerned.

#### Novel transcripts

Novel transcripts are as above but no ORF can be unambiguously assigned; these can be genuine non-coding genes or they may be partial protein-coding genes supported by limited evidence. They should be supported by at least three ESTs from independent sources (not originating from the same clone identifier).

#### Putative genes

Putative genes are identical, or have homology, to spliced ESTs but lack a significant ORF and polyA features; these are generally short two or three exon genes or gene fragments.

#### Pseudogenes

Pseudogenes (assumes no expressed evidence) have homology to proteins but generally suffer from a disrupted CDS and an active homologous gene can be found at another locus. This category can be further subdivided into processed or unprocessed pseudogenes. Sometimes these entries have an intact CDS or an open but truncated ORF, in which case there is other evidence used (for example genomic polyA stretches at the 3' end) to classify them as a pseudogene.

#### Transcribed pseudogenes

Transcribed pseudogenes are not currently given a separate tag within GENCODE and are handled by creating a pseudogene object and an overlapping transcript object with the same locus name.

#### TEC (To be experimentally confirmed)

To be experimentally confirmed (TEC) is used for non-spliced EST clusters that have polyA features. This category has been specifically created for the ENCODE project to highlight regions that could indicate the presence of novel protein coding genes that require experimental validation, either by 5' rapid amplification of cDNA ends (RACE) or RT-PCR to extend the transcripts or by confirming expression of the putatively encoded peptide with specific antibodies.

#### Artefact gene

Artefact gene is used to tag mistakes in the public databases (Ensembl/SwissProt/Trembl). Usually, these arise from high-throughput cDNA sequencing projects, which submit automatic annotation sometimes resulting in erroneous CDSs that are, for example, 3' untranslated regions (UTRs).

### GENCODE annotation of the ENCODE regions

The first release of the annotation of the 44 ENCODE regions was frozen on 29 April 2005 and was used in the E-GASP workshop. It contained 416 known loci, 26 novel CDS loci, 82 novel transcript loci, 78 putative loci, 104 processed pseudogenes and 66 unprocessed pseudogenes. The current version (release 02) was frozen on 14 October 2005. It contains 411 known loci, 30 novel CDS loci, 81 novel transcript loci, 83 putative loci, 104 processed pseudogenes and 66 unprocessed pseudogenes. The gene content has changed as a result of the experimental validation (see next section). In total, 2.9% of the nucleotides in the ENCODE regions (both strands considered separately) are covered by annotated exons (1.2% by coding and 1.7% by UTRs and non-coding), and 31% are transcribed (covered by annotated exons or introns).

Multiple transcripts are annotated at any locus where supporting evidence is available. Thus, the 487 compiled GENCODE reference loci set (compiled from coding and experimentally verified loci) corresponds to 2,608 transcripts, of which 1,097 are coding. Of the coding loci (known and novel CDS), 78% have alternative splice forms (86% of the multi-exon gene loci), with an average of 5.7 variants per locus. Of the coding variants, approximately 70% have a complete CDS (the remainder are partial); 54% of the coding loci have alternative CDS, indicating that diversity is lower at the protein level than at the transcript level as a substantial proportion of the alternative splice forms affect only the UTRs. The *RNPC2 *(RNA-binding region (RNP1, RRM) containing 2) gene has 37 variants, which is the highest number in the ENCODE regions, of which only 6 are annotated as coding.

### Experimental verification of GENCODE annotation

The initial HAVANA annotation was submitted for experimental verification (Figure 1). First, 5' RACE in 12 different tissues was employed to confirm that annotated coding genes (within both known and novel CDS locus categories) had been extended as far as possible towards the transcriptional start site, to exclude the possibility of additional exons in their 5' UTR and identify a representative full-length transcript for each locus. Of the 420 coding loci tested, 229 RACE products could be sequenced. They supported 5' extensions of 30 loci (extension of the first exon in two-thirds of the cases, new 5' exons in one-third of the cases) and new splice variants (not extending the 5' end) in 50 loci.

Second, RT-PCR in 24 tissues was used for verifying transcript (novel and putative) structures by checking the splice junctions. All 360 splice junctions in the 161 novel and putative transcript loci were tested. Of those tested, 47 loci were validated, consisting of 31 novel and 15 putative transcripts. As expected, the success rate of RT-PCR was higher for the 'novel transcripts' (37%) than for the putative transcripts (19%). Bidirectional RACE was carried out for transcript loci with successfully validated splice junctions. This supported seven loci over their full length but did not extend them.

Third, all annotated non-canonical sites (that is, all introns not conforming to the AG-GT or AG-GC rule) were tested by RT-PCR on 24 tissues. Of the annotated splice sites, 98% are canonical GT-AG and an additional 0.9% are GC-AG. There are 0.2% of AT-AC splice sites, most of them corresponding to canonical U12 introns [11]. Other non-canonical splice sites occur in the remaining 0.9% of the introns. Among 90 non-canonical splice sites tested by RT-PCR in 24 tissues, 78 reactions were negative, 11 provided other canonical junctions (most of them already annotated in other splice forms), and only 1 was confirmed (CT-TG). The very low level of success of the RT-PCRs on non-canonical splice sites in 24 tissues suggests that these events may be artifactual. As a control, we performed RT-PCR on 24 tissues (see Materials and methods) on 96 randomly selected exon pairs from within the GENCODE annotation. After sequencing of the amplimer, the annotated exon pair was confirmed in 84 cases (87%) in at least one tissue. This is essentially the expected result, given the fact that many alternative splice forms in GENCODE are likely to have a restricted expression pattern, and may not be represented in the 24 tissues tested.

**Figure 2:**
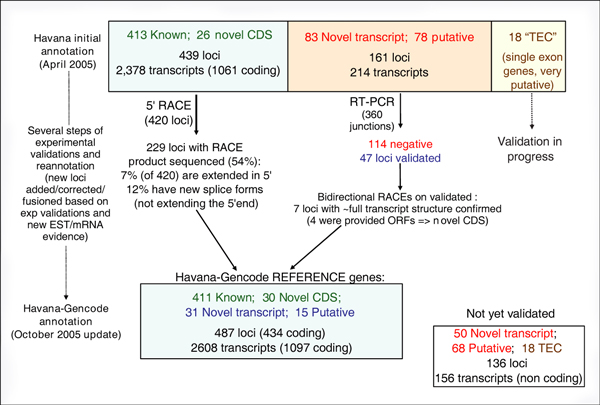
Experimental validation of HAVANA annotation. 'Known' and 'Novel_CDS' were submitted to 5' RACE, and 'Novel transcript' and 'Putative' loci were submitted to RT-PCR on all their exon junctions, followed by bi-directional RACE. Several steps of reannotation were performed during the process of experimental verification: the figure shows the update of the annotation between the first release in April 2005 and the release from October 2005.

Figure 2 summarizes the process of annotation, experimental validation and reannotation that has occurred since the original release of the GENCODE annotation in April and its current update in October 2005.

### Assessing completeness of the GENCODE annotation

To examine whether the manual annotation had missed any coding loci, RT-PCR reactions in 24 tissues were also carried out for splice junctions from all those gene objects predicted by a panel of automated gene prediction algorithms before the E-GASP workshop (Geneid [12], Genescan [13], Twinscan [14], SGP [15], Fgenesh [16], Exonify [17], Acembly [18] Ecgene [19], Ensembl EST [20]) that lie outside a HAVANA annotated gene in 13 of the 44 ENCODE regions (corresponding to the training regions for which the annotations were released before the E-GASP predictions submission deadline). Of the 1,215 exon pairs tested, only 14 (1.2%) produced a positive result, 9 of which perfectly predicted exon boundaries and 5 with displaced exon boundaries (8 other positive RT-PCRs were falling in 2 pseudogene loci). Among the 14 positive validated junctions, 8 were new splice forms internal to annotated loci, 4 were new splice forms extending annotated loci, and only 2 were completely inter-genic to any annotation. These results suggest that the GENCODE gene set was relatively complete. It was then updated to include the new splice forms/loci suggested by these experiments.

To further assess the completeness of the GENCODE annotation, we have compared it with other publicly available and widely used human gene sets: RefSeq [21] and ENSEMBL [22]. These gene sets were downloaded from the UCSC genome browser in November 2005. Table 1 shows the overlap between these sets and GENCODE by at least one bp: 99% of RefSeq, and 94% of ENSEMBL exons overlap GENCODE exons. In contrast, only 80% and 84% of the GENCODE exons overlap RefSeq and ENSEMBL exons, respectively.

**Figure 3:**
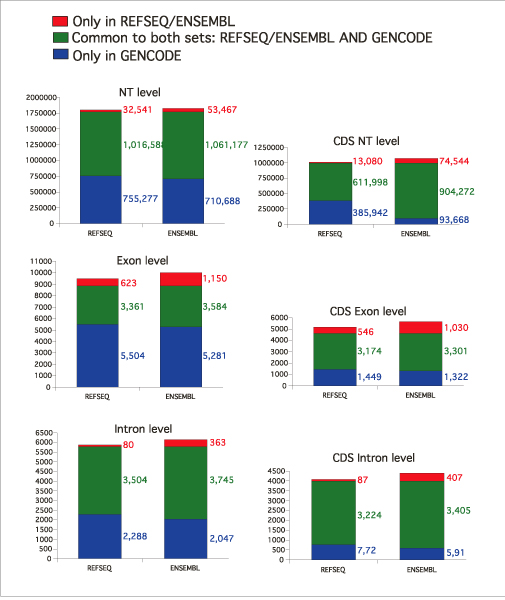
Comparison of GENCODE transcript annotation with RefSeq and ENSEMBL. The exact agreement between GENCODE and RefSeq and GENCODE and ENSEMBL exons, introns, and nucleotides (NT) for the full transcripts or only the coding parts of the transcripts (CDS) is represented: in blue is the fraction found only in GENCODE, in green the fraction common between GENCODE and the other set (RefSeq or ENSEMBL) and in red the fraction found only in the other set (RefSeq or ENSEMBL) but not in GENCODE. The RefSeq set only contained the curated transcripts tagged with the NM prefix.

Figure 3 illustrates the comparisons at exact exon/intron level. Although the exact agreement between GENCODE on the one hand, and RefSeq and ENSEMBL on the other, is lower than when considering one base overlap, the same trend is observed: 84% (3,361/3,984) of RefSeq and 76% (3,584/4,734) of ENSEMBL exons are included in the GENCODE set, but only about 40% of the GENCODE exons are included in RefSeq or ENSEMBL.

As illustrated by Figure 3, the exact agreement is larger for exons than for introns, which suggests that the disagreements are mostly found at the terminal exons, which is also reflected in the fact that the agreement is also larger for the subset of coding than for the set of all exons. In summary, the comparison shows that GENCODE contains most of the features from RefSeq and ENSEMBL but has more unique exons than the two sets, which is reflected by its high number of alternative splice forms.

### Investigation of ENCODE regions that are problematic for automatic annotation

**Figure 4:**
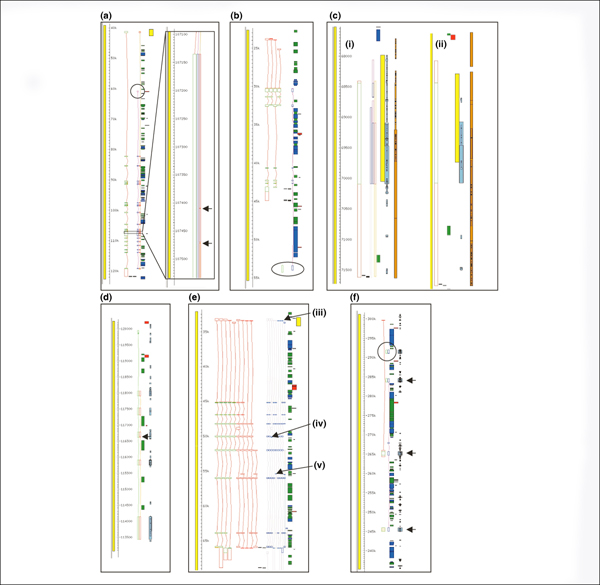
Comparison of GENCODE annotation with automated gene prediction methods. Viewed in Fmap of Acedb. Panel A shows the *MAPK1 *gene in ENr221. The GENCODE annotated gene structure is represented in green and red, the circled region highlights the different first exon identified by Pairagon (dark pink/blue) and the expanded region shows tiny introns (indicated by arrows) predicted by Ensembl (orange/red). Panel B shows the TRIM22 locus in ENm009. The structure predicted by Pairagon differs from the GENCODE structure and incorporates an unprocessed pseudogene as the final exon (circled). Panel C shows the human ANKRD43 locus in ENr221 for which AceView (light pink/blue), Pairagon and Ensembl all predict a shorter CDS than GENCODE. C ii shows the mouse ANKRD43 locus in which the upstream ATG is conserved. Panel D shows the GENCODE unprocessed pseudogene locus AC087380.14 at which Ensembl predicts a coding gene. The arrow indicates a tiny intron introduced into the prediction to splice around an in-frame premature stop codon. Panel E shows the IFNAR2 locus in ENm005 with GENCODE coding (red/green) and non-coding (all red) variants and AceView predictions. The AceView CDSs differ from GENCODE in several respects; arrow 'a' indicates several transcripts that have their CDS extended to the start of the prediction upstream of the GENCODE CDS start; arrow 'b' indicates a CDS starting in exon 5 despite the presence of an upstream ATG, which would seem to preclude (re-)initiation from this site; and arrow 'c' indicates a predicted stop codon in the fourth from last exon, which would be likely to make this transcript a target from Nonsense-mediated decay (NMD). GENCODE annotation incorporates all these variants but keeps them as transcripts as CDSs cannot be assigned with certainty. Panel F shows part of the olfactory receptor (OR) cluster in ENm009. Here Pairagon predicts a coding gene at the pseudogene locus OR52Z1P and a multi-exon gene that links separate OR loci (pseudogene locus OR51A1P, coding loci OR52A1 and OR52A5), indicated by arrows.

The gene prediction algorithms that performed most successfully in the E-GASP evaluation workshop when compared to the manual annotation were the ones that used alignments of expressed sequences to produce their gene predictions (see [7] in this issue). However, even the most successful methods of automated gene prediction achieved a maximum sensitivity of 70% at the gene level (where at least one coding transcript exon/intron structure was correctly predicted) and 45% at the transcript level (where all alternatively spliced variants were correctly predicted). There are several reasons for this. Some incidences of missed genes could be explained by the lack of high identity transcript evidence; for example, many of the olfactory receptor genes in ENm009 (Figure 4f) lack good transcript and protein support [23]. Another example is the ANKRD43 locus in ENr221, where partial coverage of the gene with human mRNA produces truncated automated predictions. However, cross-species evidence supports an extended protein-coding gene (Figure 4c). In other cases, predictors fail to make a correct prediction even though a full length transcript with perfect sequence identity is present in the databases (for example, Pairagon at the TRIM22 locus in ENm009; Figure 4b). There are also examples where the predictions differ from the manual annotation gene structure, even though they use the same supporting evidence, because of problems with automated alignment (for example, Ensembl and Pairagon at the MAP3K1 locus in ENr221; Figure 4a). A problem that appears to be associated with tandem duplicated gene clusters is the linking together of adjacent loci. The predicted transcript uses consecutive exons from more than one locus, for example for a six exon gene taking exons 1 and 2 from locus A, 3, 4 and 5 from locus B and 6 from locus C. Because the equivalent exons of the different copies of the gene are very similar (often identical), the resulting predicted transcript is an elongated structure usually covering multiple loci (for example, AceView at the HBG1/HBG2 loci in ENm009).

Another observation is that there are predictions that have an identical intron/exon structure to the manual annotation but have a different CDS. In such cases, the CDS has either a 5' extension, that is, completely matches the GENCODE CDS but uses an upstream translation initiation codon (most often non-ATG; for example, AceView at the SEPT8 locus in ENr221 and approximately 41% of AceView have a non-ATG start), or has an entirely different CDS in a different frame. The latter often results in unusual structures, with multi-exon 5' and/or 3' UTRs that are at odds with rules governing re-initiation [24] and nonsense mediated decay (NMD) [25] (for example, Pairagon at the AC008937.5 locus in ENr221 and AceView at the IFNAR2 locus in ENm005; Figure 4e). Many of the predictors suffer from reduced specificity as a result of over-prediction of CDSs at loci where manual annotation does not identify any CDS that can be confidently assigned. These fall into two types; the first includes CDS predicted at pseudogene loci, often where the pseudogene suffers from small but significant disablements (for example, Ensembl at the AC08730.14 locus in ENm009; Figure 4d); and the second includes the 'rule-breaking' types of CDSs described above (AceView at the AC008937.2 in ENr221). Almost all the predictors (with AceView the notable exception) under-predict coding (and non-coding) splice variants, most predicting one transcript per gene.

GENCODE annotation uses only primary evidence; no predictions or RefSeq entries are used to support gene structures. This has the effect of reducing the risk of propagating any errors that may be present in the databases. The gene set annotated by GENCODE is supported using evidence from all available sources, human and non-human mRNAs, ESTs and proteins. The use of non-human evidence is supported by our analysis of four exons not present in our first pass annotation identified by the UNCOVER algorithm [26], two of which are only supported by non-human EST evidence. The identification of a rare splice variant in the C16orf35 gene at the alpha globin locus is also facilitated using mouse EST evidence (J Hughes, personal communication). Importantly, manual annotation allows context to be taken into account when making a decision about difficult gene regions, which includes consulting literature and various web resources.

## Conclusion

The E-GASP workshop as part of the ENCODE project has highlighted the need for a high quality reference gene set that can be used to improve and validate prediction algorithms, as well as a scaffold for further experimentation. RT-PCR and 5' RACE of predicted exons outside the GENCODE annotation has currently not revealed additional multi-exon protein-coding genes. However, the experimental validation continually adds evidence for more splice variants. In addition, other technologies such as mapping RNA to tiling arrays [27], cap analysis gene expression (CAGE) tags [28], and gene identification signature (GIS) ditags [29] indicate there is transcriptional activity outside the regions currently annotated by the GENCODE consortium. Therefore, the annotation will be continually evolving to represent the complete transcriptional landscape of the ENCODE regions.

## Materials and methods

### Annotation pipeline and software

Before the process of manual annotation begins, an automated analysis pipeline for similarity searches and *ab initio *predictions is run. The searches are run on a computer farm and stored in an Ensembl MySQL database using a modified Ensembl analysis pipeline system [30]. All searches and prediction algorithms, except CpG island prediction (see cpgreport in the EMBOSS application suite [31]) are run on repeat masked sequence. RepeatMasker [32] is used to mask interspersed repeats, followed by Tandem repeats finder [33] to mask tandem repeats. Nucleotide sequence databases are searched with wuBLASTN [34], and significant hits are re-aligned to the unmasked genomic sequence using est2genome [35]. The Uniprot protein database [36] is searched with wuBLASTX, and the accession numbers of significant hits are looked up in the Pfam database [37]. The hidden Markov models for Pfam protein domains are aligned against the genomic sequence using Genewise [38] to provide annotation of protein domains. We also run a number of *ab initio *prediction algorithms: Genescan [13] and Fgenesh [16] for genes, tRNAscan [39] to find tRNAgenes and Eponine TSS [40], to predict transcription start sites. Annotation assessed at the E-GASP workshop used data from searches of the 24th August 2004 of dbEST, vertebrate mRNA sequences from release 80 of the EMBL nucleotide database and protein sequences from version 2.4 of Swiss-Prot/TrEMBL.

Once the automated analysis is complete, the annotator uses a Perl/Tk based graphical interface, called 'otterlace', developed in-house to edit annotation data held in a separate MySQL database system [41]. The interface displays a rich, interactive graphical view of the genomic region, showing features like database matches, gene predictions, and transcripts created by the annotators. Gapped alignments of nucleotide and protein blast hits to the genomic sequence are viewed and explored using the 'Blixem' alignment viewer [42]. Additionally, the 'Dotter' dot plot tool [42] is used for showing the pair-wise alignments of unmasked sequence, thus revealing the location of exons that are occasionally missed by the automated blast searches because of their small size and/or match to repeat-masked sequence. The interface provides a number of tools that the annotator uses to build genes and edit annotations: adding transcripts, exon coordinates, translation regions, gene names and descriptions, remarks and polyadenlyation signals and sites.

### Rapid amplification of cDNA ends

Both 5' and 3' RACE were performed on 12 human poly(A)^+ ^RNAs (brain, heart, kidney, spleen, liver, colon, small intestine, muscle, lung, stomach, testis, placenta) using the BD SMART™ RACE cDNA amplification kit (BD BioScience-Clontech Catalogue No.634914, Mountain View, CA 95043, USA). Double-stranded cDNA synthesis and adaptor ligations to the synthesized cDNA were done according to the manufacturer's instructions. RACE fragments were separated on agarose gels and one or two strong single bands per gene purified and sequenced directly. Thus, successful RACE reactions appearing as a smear on the agarose gel would be discarded, therefore producing an approximate 54% success rate.

### RT-PCR

Similar amounts of 24 human cDNAs (brain, heart, kidney, spleen, liver, colon, small intestine, muscle, lung, stomach, testis, placenta, skin, PBLs, bone marrow, fetal brain, fetal liver, fetal kidney, fetal heart, fetal lung, thymus, pancreas, mammary glands, prostate; final dilution 1,000×) were mixed with JumpStart REDTaq ReadyMix (Sigma, St Louis, MO, USA) and 4 ng/μl primers (Sigma-Genosys, St Louis, MO, USA)) with a BioMek 2000 robot (Beckman, Fullerton, CA, USA) as described and modified [43-45]. The 10 first cycles of PCR amplification were performed with a touchdown annealing temperature decreasing from 60°C to 50°C; the annealing temperature of the next 30 cycles was 50°C. Amplimers were separated on 'Ready to Run' precast gels (Pfizer, New York, NY, USA) and sequenced. This procedure was used to experimentally assay 1,215 exon-exon junctions of human genes predicted by five *ab initio *and four EST-based methods outside of HAVANA objects and 83 HAVANA novel and 78 putative transcripts (see Results and discussion for details).

## Figures and Tables

**Table 1:** Analysis of RefSeq and ENSEMBL ENCODE annotation compared with GENCODE

	RefSeq	ENSEMBL
No. (unique) exons	3,984	4,734
No. transcripts	577	738
No. exons overlapping GENCODE exons (%)	5,118 (98.6%)	4,469 (94.4%)
No. transcripts overlapping GENCODE (%)	567 (98.3%)	675 (91.5%)
No. GENCODE exons overlapped (total = 8,865) (%)	7,084 (80.0%)	7,450 (84.0%)
No. GENCODE transcripts overlapped (total = 2,608) (%)	2,327 (89.2%)	2,395 (91.8%)
